# Supplementary material for: Identifying the suite of genes central to swimming in the biocontrol bacterium Pseudomonas protegens Pf-5
Source: Microb Genom. 2024 Mar 28;10(3):001212. doi: 10.1099/mgen.0.001212 (PMC11004494; doi:10.1099/mgen.0.001212)
Supplement: Uncited Supplementary Material 1. [file mgen-10-01212-s001.pdf]

Supplementary information for

Identifying the suite of genes central to swimming in the biocontrol bacteria *Pseudomonas protegens* Pf-5

Fabian BK, Foster C, Asher A, Hassan KA, Paulsen IT[#] and Tetu SG[#]

[#]Address correspondence to Sasha Tetu, sasha.tetu@mq.edu.au and Ian Paulsen, ian.paulsen@mq.edu.au.

This PDF file includes:

Table S1
Table S2
Table S3
Figure S1
Figure S2
Figure S3
Figure S4

Other supplementary materials for this manuscript include:

Dataset S1

Supplementary tables

Table S1. *Pseudomonas protegens* Pf-5 transposon mutant library metrics from Bio-Tradis pipeline analysis.

Treatment	Replicate	Total no. of reads	No. (%) of reads with matching transposon tag	No. (%) of aligned reads	No. of unique insertion sites	Average distance between unique insertion sites (bp)
Swimming	1	1,363,792	1,359,791 (99.7%)	1,333,222 (98.0%)	306,381	23.1
Swimming	2	1,758,995	1,753,807 (99.7%)	1,718,672 (98.0%)	322,915	21.9
Control	1	3,951,744	3,922,215 (99.3%)	2,187,032 (55.8%)	352,775	20.1
Control	2	2,790,059	2,770,318 (99.3%)	1,941,521 (70.1%)	345,809	20.5

Table S2. Primers for allelic exchange mutagenesis of genes. The random six nucleotides are italicised, restriction enzyme recognition sites are indicated in bold, and the complementary regions between the UpR/DnF primers are underlined.

Genes	Amplification regions	Primer names	Primer sequences (5'-3')	Amino acids remaining (incl start & stop codon)
PFL_6108	5' flanking	6108-UpF-XbaI	*CGTGCT***TCTAGA**GGAGGTCAGTCGGATTTCGC	10
		6108-UpR	GGCCTTGGTGGAGCTTCTGCCAACCATGCTTG	
	3' flanking	6108-DnF	GTTGGCAGAAGCTCCACCAAGGCCTGACGG	
		6108-DnR-XbaI	*GTGAGG***TCTAGA**CGTAGCTGTCCTGCTCGAAG	
PFL_6109	5' flanking	6109-UpF-XbaI	GTGAGG**TCTAGA**ATCATCATGCTCACCGCCAA	18
		6109-UpR	GGTCAGGCTCTGAATCAGGGTGCCATGCCAGT	
	3' flanking	6109-DnF	GGCACCCTGATTCAGAGCCTGACCGACCAG	
		6109-DnR-XbaI	*ATGACG***TCTAGA**GACCACGATGTGCAGGTAGG	
PFL_6119	5' flanking	6119-UpF-XbaI	*ATGACG***TCTAGA**GCTCTTCCAGGGAGAACATC	9
		6119-UpR	GGCATGAAACTGCTGAAAGAAGGCGCATAAGC	
	3' flanking	6119-DnF	GCCTTCTTTCAGCAGTTTCATGCCTTACTCCT	
		6119-DnR-XbaI	*GTGAGG***TCTAGA**GTTCGGCAGCGTCTTGTTAC	

Table S3. Primers for verification of gene deletions via PCR and sequencing. These primers anneal external to the gene deletion region of the genome, or within the vector backbone external to the cloned insert region.

Genes	Functions	Primer names	Primer sequences (5'-3')
PFL_6108	Verification of Δ*phoB* mutant	6108ExtF	CGTCAAGCAAGGCCCTATGG
		6108ExtR	TTGCGCATCTGTTCCAGCT
PFL_6109	Verification of Δ*phoR* mutant	6109ExtF	GACCTGATCCTGCTGGACTG
		6109ExtR	GGTAGATGGCCGGGTACATC
PFL_6119	Verification of Δ*pstS* mutant	6119ExtF	GGATCGGCAGGTCGACTTTG
		6119ExtR	ACAGCCTGGCCATTATCTTCC
pEx18Tc vector	Verification of mutant allele constructs	pEX18Tc_F	CCTCTTCGCTATTACGCCAG
		pEX18Tc_R	GTTGTGTGGAATTGTGAGCG

Supplementary figures

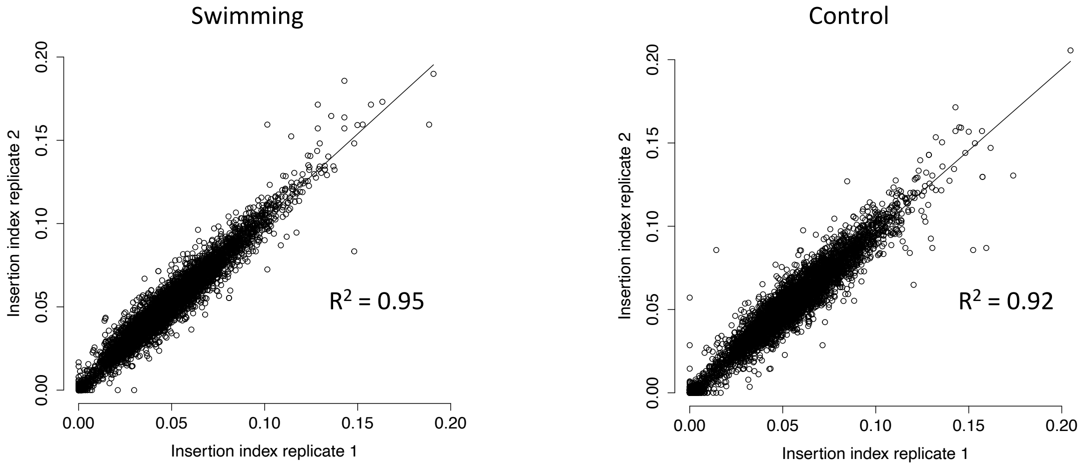

Figure S1. Correlation of gene insertion indexes for the two replicates of the *Pseudomonas protegens* Pf-5 mutant library in swimming conditions and the control. Insertion index is calculated as the number of transposon insertion sites in a gene divided by the gene length. Figure generated using R (1).

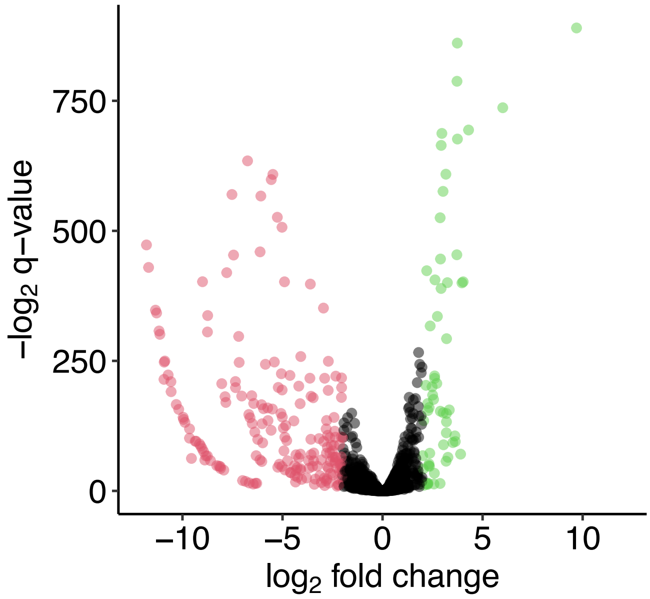

Figure S2. Volcano plot showing log_2-fold change of all *Pseudomonas protegens* Pf-5 genes from swimming assay when compared with the control. Points in red have a significant fold change < -2 ($p < 0.01$); loss of these genes is detrimental for swimming fitness. Points in green have a significant fold change > 2 ($p < 0.01$); loss of these genes is beneficial for swimming fitness. Figure generated using the R package ggplot2 (2).

Phosphate concentration	Parental strain	Δ*phoB*	Δ*phoR*	Δ*pstS*
6.6 mM	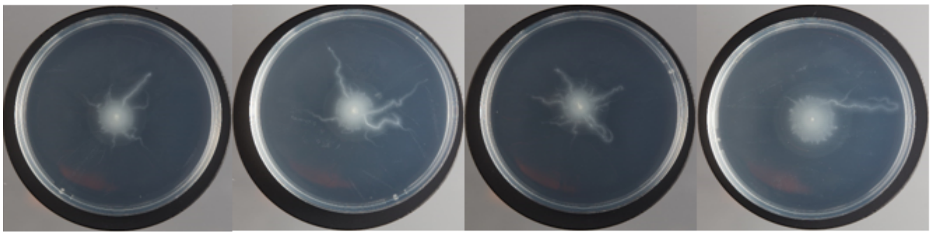			
0.05 mM	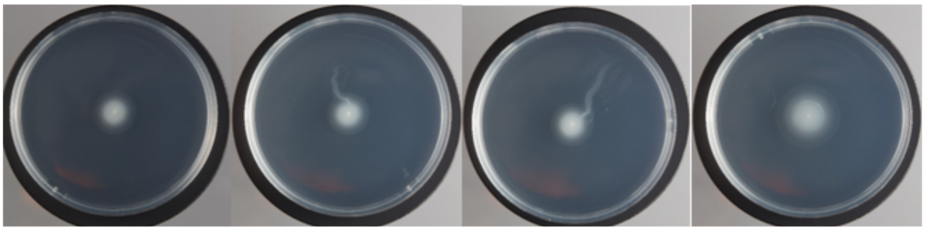			

Figure S3. Representative swimming assay plates for *Pseudomonas protegens* Pf-5 parental strain and *pstS*, *phoB* and *phoR* single knockout mutants after 24 hours growth at 22°C on BM2 0.3% agar plates with 0.05 or 6.6 mM phosphate.

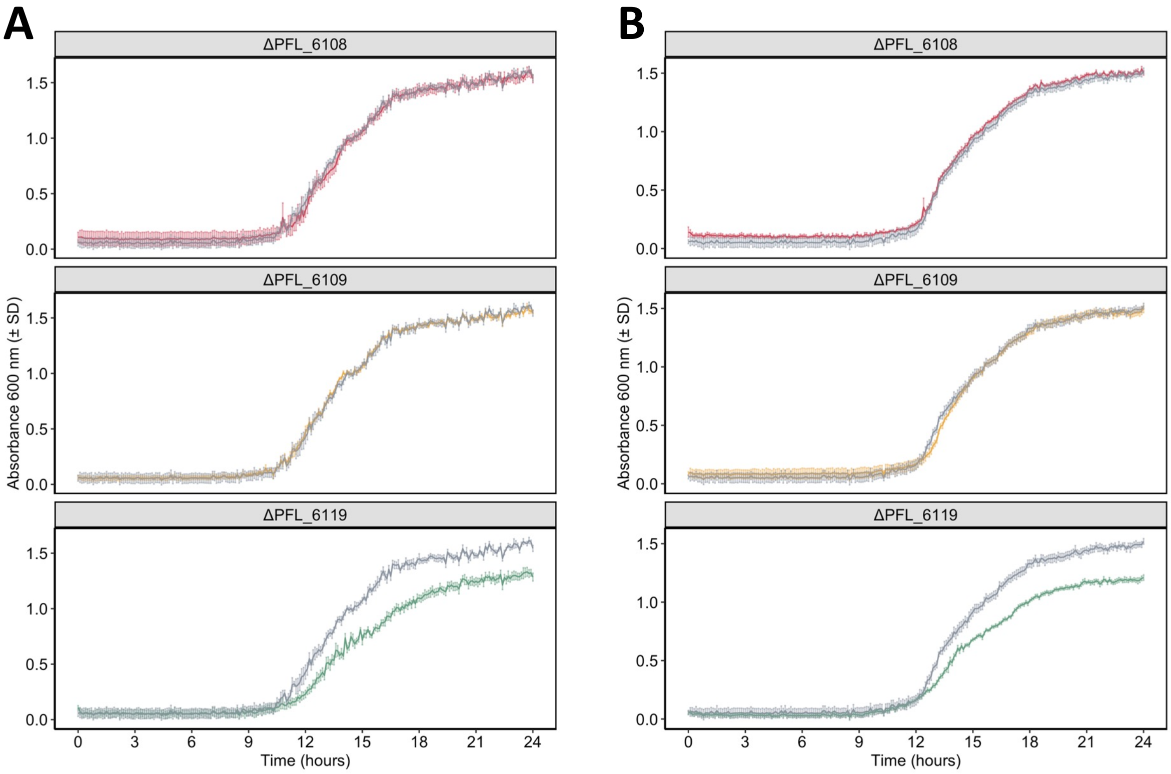

Figure S4. Growth of *Pseudomonas protegens* Pf-5 single knockout mutants compared to wild-type (grey) on (A) BM2 broth + 6.6 mM phosphate and (B) BM2 broth + 0.05 mM phosphate. Absorbance was measured at 600 nm, four replicates per strain were used and the mean absorbance (± SD) is shown. Figure generated using the R package ggplot2 (2).

Legend for Dataset S1

Dataset S1. Fold changes of all *Pseudomonas protegens* Pf-5 genes from comparing the output pool (motile cells) to the control pool. Metadata located in separate tab.

Supplementary references

1. R Core Team. 2022. R: A language and environment for statistical computing, R Foundation for Statistical Computing, Vienna, Austria.
2. Wickham H. 2016. ggplot2: Elegant Graphics for Data Analysis, Springer-Verlag, New York.
